# An Efficient One-Pot Three-Component Synthesis of Fused 1,4-Dihydropyridines Using HY-Zeolite

**DOI:** 10.3390/molecules14041468

**Published:** 2009-04-08

**Authors:** Mohammad Nikpassand, Manouchehr Mamaghani, Khalil Tabatabaeian

**Affiliations:** Department of Chemistry, Faculty of Sciences, University of Guilan, P. O. Box 41335-1914, Rasht, Iran; E-mails: nikpassand@guilan.ac.ir (M.N.), taba@guilan.ac.ir (K.T.)

**Keywords:** 1,4-Dihydropyridine, Dimedone, NH_4_OAc, One-Pot Conversion, HY-Zeolite.

## Abstract

A facile and convenient protocol was developed for the fast (2.5-3.5 h) and high yielding (70-90 %) synthesis of fused 1,4-dihydropyridines from dimedone in the presence of HY-zeolite as an efficient recyclable heterogeneous catalyst.

## 1. Introduction

1,4-Dihydropyridines represent an important class of compounds which are found in many biologically active products, such as vasodilator, bronchodilator, anti-atherosclerotic, antitumor, geroprotective, hepatoprotective and antidiabetic agents [1]. Numerous synthetic methods have been reported for the preparation of 1,4-dihydropyridine derivatives under classical or modified conditions [2,3,4,5,6,7,8,9,10]. However, some of these methods suffer from long reaction times, low yields, use of large quantities of volatile organic solvents, harsh reaction conditions and tedious workups, therefore, development of an efficient and versatile method is still required. 

HY-zeolite is unique acid heterogeneous catalyst that has become popular over the last two decades. It is used in various chemical transformations, such as liquid phase acylation of amines [11], direct conversion of aldehydes into amines [12], selective removal of *N*-Boc protecting groups from aromatic amines [13], one-pot synthesis of 2,3-dihydro-2,2-dimethylbenzofurans [14], and one-pot syntheses of polyhydroquinolines [15].

This remarkable catalytic activity together with easy availability, operational simplicity and recoverability of HY-zeolite encouraged us to utilize this catalyst for the synthesis of fused 1,4-dihydropyridine derivatives.

## 2. Results and Discussion

**Scheme 1:**
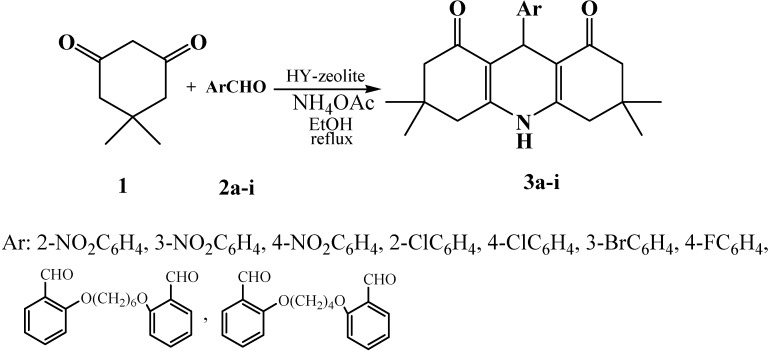
Synthesis of 1,4-dihydropyridines from dimedone in the presence of HY-zeolite.

Recent developments in 1,4-dihydropyridine chemistry and our continued interest in the development of efficient and environmentally friendly procedures for the synthesis of heterocyclic compounds [16,17,18,19,20,21,22,23], prompted us to study the conversion of dimedone into fused 1,4-dihydropyridines in the presence of HY zeolite (Si/Al: 2.54). The reaction of dimedone (**1**, 2 eq.) with 1 equiv. of each of various arylaldehydes **2a-i** and NH_4_OAc in EtOH in the presence of HY-zeolite furnished the desired fused 1,4-dihydropyridine derivatives **3a-i** (Scheme 1) in reasonable reaction times (2.5-3.5 h) and high yields (70-90%) (Table 1). Moreover, the catalyst was easily recovered and the high catalytic activity was maintained even after third reuse of the catalyst. 

The reaction, using substrate **2a**, was also performed in the presence of CH_3_CO_2_H (1 mL/mmol substrate, 5 h, 67%), ZnCl_2_ (0.01 g/mmol substrate, 6 h, 65%) and RuCl_3_ (0.01 g/mmol substrate, 5.5 h, 70%) under optimized condition, which furnished the desired product after longer reaction times and in lower yields. The control reaction was carried out on the substrates **2a** and 2d in refluxing ethanol without the zeolite catalyst. Under this classical condition the reaction proceeded smoothly resulting in the expected 1,4-dihydropyridine products after longer reaction times and in lower yields (**2a**: 6.5 h, 65%; **2d**: 8.0 h, 63%). These results revealed that using HY-zeolite as catalyst appreciably shortens the reaction times and increases the product yields. All of the products were fully characterized by spectroscopic methods (IR, ^1^H-NMR, ^13^C-NMR) and elemental analysis.

**Table 1:** Synthesis of fused 1,4-dihydropyridine derivatives (**3a-i**) using HY-zeolite.

Entry	Aldehyde	Time (h)	Yield (%)^1,2^
**a**	2-NO_2_ C_6_H_4_CHO	2.5 (6.5)^3^	75 (65)^3^
**b**	3-NO_2_ C_6_H_4_CHO	2.5	83
**c**	4-NO_2_ C_6_H_4_CHO	3.0	79
**d**	2-ClC_6_H_4_CHO	3.5 (8.0)^3^	75 (63)^3^
**e**	4-ClC_6_H_4_CHO	3.5	77
**f**	3-BrC_6_H_4_CHO	3.0	90
**g**	4-FC_6_H_4_CHO	3.5	70
**h**	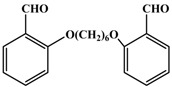	3.5	82
**i**	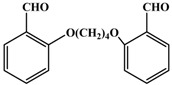	3.5	78

^1^ Isolated yields; ^2^ All the products were Identified by spectroscopic (IR, ^1^H-NMR, ^13^C- NMR) and elemental analyses. ^3^A mixture of dimedone (20 mmol), aryl aldehydes (**2a**, **2d**) (10 mmol) and NH_4_OAc (10 mmol) in EtOH (10 mL) was refluxed for the required reaction time, after which removal of the solvent produced the desired products **3a** and **3d **in 65% and 63% yields, respectively. 

## Conclusions

In summary, we report a simple protocol for the synthesis of fused 1,4-dihydropyridines using HY zeolite as an efficient catalyst. The simplicity, easy workup, together with the use of inexpensive, environmentally friendly and reusabile catalyst, are the notable features of this catalytic procedure.

## 3. Experimental

### 3.1. General

Melting points were measured on an Electrothermal 9100 apparatus. IR spectra were determined on a Shimadzo IR-470 spectrometer. ^1^H-NMR and ^13^C-NMR spectra were recorded on a 500 MHz Bruker DRX-500 in CDCl_3_ as solvent and with TMS as internal standard. Chemicals were purchased from Merck and Fluka. Elemental analyses were done on a Carlo-Erba EA1110CNNO-S analyzer and agreed with the calculated values. All solvents used were dried and distilled according to standard procedures.

### 3.2. General procedure for the synthesis of 3a-i in the presence of HY-zeolite

A mixture of dimedone (20 mmol), aryl aldehydes (10 mmol), NH_4_OAc (10 mmol), HY-zeolite (0.1 g) in EtOH (10 mL) was refluxed for the required reaction time (Table 1). The progress of the reaction was monitored by TLC (EtOAc: petroleum ether 3:1). After completion of the reaction, the mixture was cooled to room temperature and filtered. The filtrate was concentrated under vacuum and the residue was recrystallized from ethanol to produce 1,4-dihydropyridine derivatives **3a-i** as pure crystalline products in 70-90% yields. 

*3,3,6,6-Tetramethyl-9-(2-nitrophenyl)-3,4,6,7-tetrahydroacridine-1,8(2H,5H,9H,10H)-dione* (**3a**): Brown solid, mp 281-282 ^o^C; ^1^H-NMR δ 0.96 (s, 6H), 1.06 (s, 6H), 2.23- 2.46 (m, 8H), 5.80 (s, 1H), 7.21 (t, *J* = 6.99 Hz, 1H), 7.30- 7.35 (d, *J* = 6.81 Hz, 1H), 7.41- 7.48 (m, 2H); ^13^C-NMR δ 27.8, 29.5, 32.9, 41.2, 51.1, 112.9, 124.5, 127.0, 132.5, 134.2, 141.2, 149.6, 149.8, 195.9; IR (neat, cm^-1^) 3450, 3080, 2980,1650, 1520, 1480, 1360, 1220, 1140. Anal. Calcd. for C_23_H_26_N_2_O_4_: C, 70.03; H, 6.63; N, 7.10. Found: C, 70.25; H, 6.48; N, 7.02. 

*3,3,6,6-Tetramethyl-9-(3-nitrophenyl)-3,4,6,7-tetrahydroacridine-1,8(2H,5H,9H,10H)-dione* (**3b**): Off white solid, mp 273-275 ^o^C; ^1^H-NMR δ 0.86 (s, 6H), 1.01 (s, 6H), 2.0 (d, *J* = 16.3 Hz , 2H ), 2.10 (d, *J* = 16.3 Hz , 2H), 2.28 (d, *J* = 17.1 Hz, 2H), 2.35 (d, *J* = 17.1 Hz, 2H), 5.01 (s, 1H), 7.29 (t, *J* = 7.86 Hz, 1H), 7.65 (d, *J* = 6.9 Hz, 1H), 7.83 (d, *J* = 8.1 Hz, 1H), 8.02 (s, 1H), 8.90 (s, br., 1H) ; ^13^C-NMR δ 27.4, 29.9, 32.9, 34.4, 51.1, 112.2, 121.2, 123.1, 130.0, 135.2, 148.4, 149.6, 150.2, 195.6; IR (neat, cm^-1^) 3380, 3060, 2960, 1645, 1610, 1520, 1480, 1360, 1340, 1220, 1140. Anal. Calcd. for C_23_H_26_N_2_O_4_: C, 70.03; H, 6.63; N, 7.10. Found: C, 69.88; H, 6.52; N, 7.28. 

*3,3,6,6-Tetramethyl-9-(4-nitrophenyl)-3,4,6,7-tetrahydroacridine-1,8(2H,5H,9H,10H)-dione* (**3c**): Yellow-orange solid, mp 282-283 ^o^C; ^1^H-NMR δ 0.87 (s, 6H), 1.02 (s, 6H), 2.04 (d, *J* = 16.3 Hz, 2H), 2.15 (d, *J* = 16.3 Hz, 2H), 2.26 (d, *J* = 17.0 Hz, 2H), 2.34 (d, *J* = 17.0 Hz, 2H), 5.05 (s, 1H), 7.44 (d, *J* = 8.5 Hz, 2H), 7.98 (d, *J* = 6.9 Hz, 2H), 8.49 (s, 1H); ^13^C-NMR δ 27.4, 29.9, 32.9, 34.9, 51.0, 112.3, 123.5, 129.4, 146.3, 149.8, 154.9, 195.6; IR (neat, cm^-1^) 3384, 3070, 2956,1643, 1515, 1479, 1342, 1218, 1166. Anal. Calcd. for C_23_H_26_N_2_O_4_: C, 70.03; H, 6.63; N, 7.10. (Found: C, 69.93; H, 6.75; N, 7.32. 

*9-(2-Chlorophenyl)-3,3,6,6-tetramethyl-3,4,6,7-tetrahydroacridine-1,8(2H,5H,9H,10H)-dione* (**3d**): Off white solid, mp 217-219 ^o^C; ^1^H-NMR δ 1.05 (s, 6H), 1.14 (s, 6H), 2.10-2.30 (m, 4H), 2.45-2.62 (m, 4H), 5.04 (s, 1H), 7.09 (t, *J*= 7.2 Hz, 1H), 7.01-7.19 (m, 1H), 7.25 (d, *J* = 7.5 Hz, 1H), 7.47 (d, *J* = 6.4 Hz, 1H); ^13^C-NMR δ 27.8, 29.7, 32.5, 41.2, 51.2, 114.2, 126.8, 127.6, 128.2, 130.6, 133.9, 149.4, 163.5, 197.0; IR (neat, cm^-1^) 3400, 2980,1660, 1620, 1465, 1350, 1200. Anal. Calcd. for C_23_H_26_ClNO_2_: C, 71.96; H, 6.82; N, 3.65. Found: C, 71.72; H, 6.55; N, 3.81.

*9-(4-Chlorophenyl)-3,3,6,6-tetramethyl-3,4,6,7-tetrahydroacridine-1,8(2H,5H,9H,10H)-dione* (**3e**): Off white solid, mp 228-229 ^o^C; ^1^H-NMR δ 1.03 (s, 6H), 1.15 (s, 6H), 2.17- 3.33 (m, 8H), 5.51 (s, 1H), 7.20 (d, *J* = 8.3 Hz, 2H), 7.32 (d, *J* = 8.3 Hz, 2H), 8.06 (s, 1H); ^13^C-NMR δ 27.5, 30.0, 33.0, 41.1, 51.2, 113.3, 128.5, 129.9, 132.0, 145.6, 149.7, 196.8; IR (neat, cm^-1^) 3429, 3178, 3060, 2958,1641,1606, 1488, 1222, 1143. Anal. Calcd. for C_23_H_26_ClNO_2_ : C, 71.96; H, 6.82; N, 3.65. Found: C, 71.83; H, 6.71; N, 3.55. 

9-(3-Bromophenyl)-3,3,6,6-tetramethyl-3,4,6,7-tetrahydroacridine-1,8(2H,5H,9H,10H)-dione (**3f**): Off white solid, mp 305-307 ^o^C; ^1^H-NMR δ 1.01 (s, 6H), 1.12 (s, 6H), 2.18-2.35 (m, 8H), 5.05 (s, 1H), 7.11 (t, *J* = 7.74 Hz, 1H), 7.25 (d, *J* = 7.73 Hz, 1H), 7.35 (t, *J* = 7.59 Hz, 1H), 7.48 (s, 1H), 7.98 (s, 1H); ^13^C-NMR δ 27.5, 30.0, 33.1, 41.1, 51.3, 113.1, 122.6, 127.5, 129.5, 130.0, 131.4, 149.3, 149.8, 196.3; IR (neat, cm^-1^) 3280, 3080, 2970, 1640, 1555, 1250, 1220. Anal. Calcd. for C_23_H_26_BrNO_2_: C, 64.49; H, 6.11; N, 3.27. Found: C, 64.66; H, 6.32; N, 3.11. 

*9-(4-Fluorophenyl)-3,3,6,6-tetramethyl-3,4,6,7-tetrahydroacridine-1,8(2H,5H,9H,10H)-dione* (**3g**): Yellow solid, mp 215-218 ^o^C; ^1^H-NMR δ 0.98 (s, 6H), 1.10 (s, 6H), 2.04- 2.38 (m, 8H), 5.09 (s, 1H), 6.90 (m, 2H), 7.30 (m, 2H), 8.30 (s, 1H); ^13^C-NMR δ 27.4, 30.0, 33.0, 41.0, 51.3, 113.3, 115.2, 129.7, 143.0, 149.9, 162.5, 196.5 ; IR (neat, cm^-1^) 3355, 3047, 2958, 1618, 1498, 1362, 1220, 1147. Anal. Calcd. for C_23_H_26_FNO_2_: C, 75.18; H, 7.12; N, 3.81. Found: C, 75.32; H, 7.23; N, 3.67. 

*3,3,6,6-tetramethyl-9-(2-(6-(2-(3,3,6,6-tetramethyl-3,4,6,7-tetrahydroacridine1,8(2H,5H,9H,10H)-dione-9-yl)hexoxy)phenoxy)phenyl-3,4,6,7-tetrahydroacridine-1,8(2H,5H,9H,10H)-dione* (**3h**): Off white solid, mp 275-277 ^o^C; ^1^H-NMR δ 0.90 (s, 12H), 0.97 (s, 12H), 1.01-1.06 (m, 4H), 1.96-2.29 (m, 20H), 3.97 (s, br., 4H), 5.30 (s, 2H), 6.71-7.26 (m, 8H), 8.83 (s, 2H); IR (neat, cm^-1^) 3290, 3065, 2960, 1645, 1490, 1365, 1225, 745. Anal. Calcd. for C_52_H_64_N_2_O_6_: C, 76.81; H, 7.93; N, 3.45. Found: C, 76.95; H, 7.82; N, 3.59. 

*3,3,6,6-tetramethyl-9-(2-(4-(2-(3,3,6,6-tetramethyl-3,4,6,7-tetrahydroacridine1,8(2H,5H,9H,10H)-dione-9-yl)butoxy)phenoxy)phenyl-3,4,6,7-tetrahydroacridine-1,8(2H,5H,9H,10H)-dione* (**3i**): Off white solid, mp 269-271 ^o^C; ^1^H-NMR δ 0.92 (s, 12H), 1.04 (s, 12H), 1.98-2.35 (m, 20H), 4.06 (s, br., 4H), 5.22 (s, 2H), 6.80-7.06 (m, 8H), 8.67 (s, 2H); IR (neat, cm^-1^) 3290, 3060, 2960, 1660, 1480, 1360, 1235, 745. Anal. Calcd. for C_50_H_60_N_2_O_6_: C, 76.50; H, 7.70; N, 3.57. Found: C, 76.38; H, 7.78; N, 3.60.
